# Personalized Digital Care Pathways Enable Enhanced Patient Management as Perceived by Health Care Professionals: Mixed-Methods Study

**DOI:** 10.2196/68581

**Published:** 2025-05-15

**Authors:** David Rodrigues, Clara Jasmins, Ricardo Ladeiras-Lopes, Luis Patrao, Eduardo Freire Rodrigues

**Affiliations:** 1 Comprehensive Health Research Centre, NOVA Medical School, Lisbon, Portugal; 2 Family Medicine Department, NOVA Medical School, Lisbon, Portugal; 3 UpHill Health, SA, Lisbon, Portugal; 4 Cardiovascular R&D Centre-UnIC@RISE, Department of Surgery and Physiology, Faculty of Medicine, University of Porto, R. Dr. Plácido da Costa, Porto, 4200-450, Portugal, 351 966897374; 5 Faculty of Health Sciences, University of Beira Interior, Covilhã, Portugal

**Keywords:** care integration, clinical pathway, digital, heart failure

## Abstract

**Background:**

Clinical decision support systems are known to improve adherence to clinical practice guidelines and patient outcomes by providing clinicians with timely, accurate, and appropriate knowledge.

**Objective:**

This study investigates the perceived usefulness and practical implementation of UpHill Route v3, a personalized digital care pathway (PDCP) system, in enhancing clinical decision-making and patient management across various clinical settings.

**Methods:**

A mixed-methods retrospective study was conducted among medical doctors and nurses from four National Health System-Local Health Units in Portugal. Data were collected from May 2023 to April 2024. The primary data source was an anonymous questionnaire assessing health care professionals’ perceptions of UpHill Route v3’s usefulness using the Likert scale ranging from 0 (do not agree) to 10 (totally agree). Secondary analysis involved quantifying decisions across heart failure, multimorbidity, diabetes, and colorectal and breast cancer clinical pathways. These data were collected from user interactions with UpHill Route v3 as well as from its internal database. Descriptive and bivariate statistics were used to analyze the data.

**Results:**

A total of 22 health care professionals with mean age 44.7 (SD 10.6) years, including 15 (68%) female participants and 9 (41%) physicians were included in the study. High ratings for adherence to clinical protocols, mean score 8.06 (SD 1.73); clinical decision support, mean score 8.05 (SD 1.73); patient care improvement, mean score 7.63 (SD 2.22); and confidence in patient management, mean score 8.26 (SD 1.56) were reported. Secondary analysis showed that across 3574 patients, 25,741 clinical decisions were informed, and 9254 actions were performed with the assistance of the PDCP tool.

**Conclusions:**

The UpHill Route v3 PDCP tool is highly valued by health care professionals for its ability to support clinical decision-making and improve operational efficiency across various clinical settings. Our findings suggest that this tool can effectively bridge the gap between clinical guidelines and real-world practice.

## Introduction

Computerized clinical decision support systems (CDSS) are designed to enhance patient care by providing clinicians and patients with timely, accurate, and appropriate knowledge in the context of specific medical interactions or encounters. These systems have gained significant traction for their potential to improve adherence to clinical practice guidelines, bridging the gap between knowledge and its application in diverse clinical contexts [1,2]. Systematic reviews and studies have been conducted to evaluate the effectiveness of CDSS, showing that CDSS can lead to improvements in process adherence, thus leading to higher diagnosis accuracy and comprehensive disease management [3,4]. There are multiple types of CDSS. Traditional CDSS primarily offer static recommendations, whereas personalized CDSS integrates patient-specific data from an electronic health record (EHR) to tailor clinical recommendations. Personalized digital care pathway (PDCP) represents a further advancement, extending CDSS capabilities beyond single encounters by ensuring continuity of care across multiple medical interactions. PDCP systems facilitate decision-making and action execution across multidisciplinary teams, while maintaining compliance with best practice protocols and continuously adapting based on patient-specific data. These systems have demonstrated benefits for patients, health care professionals (HCP), and institutions by contributing to improved service quality, workforce efficiency, and patient outcomes. This expands the scope of CDSS to the entire continuum of care, enabling the interaction among multiple multidisciplinary teams through a shared care pathway. [4,5].

PDCPs ensure that each HCP delivers tailored care aligned with standard protocols and guidelines, especially relevant for chronic conditions requiring frequent assessments and timely therapy adjustments across different contexts [6]. Current management strategies for chronic patient care typically rely on traditional methods such as face-to-face consultations, periodic monitoring, and manual documentation of patient records. Care plans are often manually developed based on clinical guidelines and personal clinical judgment, with minimal decision support integration. Most EHRs lack advanced decision support capabilities, decision characterisation, and patient communication features. Overall, current management strategies can be time-consuming and prone to variability in care delivery.

For example, CDSS have been shown to improve patient outcomes in heart failure management by enhancing monitoring, management, and optimization of therapies [7]. As more clinical practice guidelines emphasize the necessity of digital solutions for early disease detection and patient self-care [8], multidisciplinary chronic disease management programs powered by CDSS tools have yielded better quality of life, lower readmission rates, and reduced mortality [3]. Despite these benefits, there is a lack of evidence regarding the perceived usefulness of CDSS among HCPs using these systems and on the quantification and characterization of this usage on the number and types of decisions and actions usually performed by CDSS in chronic disease management [3,9].

This study hypothesizes that HCPs consider PDCPs, such as Uphill Route v3, to be more valuable than current management strategies for chronic patient management. As a PDCP and a subtype of CDSS, UpHill Route v3 aids medical decision-making and improves patient management by increasing compliance with locally validated clinical pathways. The software creates personalized care pathways by capturing patient health and demographic data from hospital EHRs, manual inputs by HCPs, or self-reported patient questionnaires (sent via text messages, web forms, or voice interaction capabilities). This approach further supports HCP clinical decisions and tasks throughout the clinical pathway. Thus, the main aim of this study was to characterize the perceived usefulness of a PDCP in chronic patient management. The secondary aim was to describe the number and types of decisions supported by the software, thus quantifying them as a proxy for the system’s impact on decision support and patient management efficiency.

## Methods

### Study Design

This was a mixed-methods retrospective study, conducted to evaluate the usefulness of PDCP as a CDSS for HCPs. For the primary aim, qualitative data was collected through a questionnaire directed at HCPs using UpHill Route v3, to assess the perceived usefulness of PDCP and compare it with current management practices for chronic patient care. For the secondary aim, quantitative data on decisions and tasks performed during care pathways was retrieved from UpHill Route v3. This design allowed for a comprehensive understanding of the impact of UpHill Route v3 on clinical decision-making and patient management.

### Setting

Medical doctors and nurses from four National Health System Local Health Units (LHUs) in Portugal, who were pilot-testing UpHill Route v3, were invited to participate. These professionals were trained in using UpHill Route v3 as part of the software implementation framework, as described in the software technical documentation and instructions for use. As part of the implementation process, clinical content embedded in the software was validated and approved, meaning that clinical leaders from the enrolled institutions approved the protocols reflected on the software.

The inclusion of patients in UpHill Route v3 was the exclusive responsibility of HCPs, who were instructed and trained to use the software in accordance with the technical documentation for noncritical patients in non-critical clinical settings.

The study encompassed the central, capital, and southern coastal regions of Portugal and conducted from May 2023 to April 2024. The LHUs—Litoral Alentejano, Amadora Sintra, Coimbra, and São José—serve a combined population of over 1.40 million people across 17,660 km². This vast region includes urban and suburban populations, and the clinical journeys in these four LHUs include heart failure, multimorbidity (ie, combined heart failure, chronic obstructive pulmonary disease, and diabetes), diabetes, and colorectal and breast cancer, which represent a diverse spectrum of chronic diseases with frequent acute care interventions.

### Participants

To be eligible for this study, participants were required to be part of the HCP user base at the previously described health units that were pilot testing UpHill Route v3 in their daily practice and to have completed the training sessions in the proper use of the software. The instruction to use UpHill Route v3 only in noncritical settings was directly related to the targeted diseases, which were chronic or elective conditions integrated into predefined care pathways. The HCPs were briefed on the appropriate use of the system, and adherence to this criterion was monitored through participant feedback and usage data.

It is important to note that the device does not autonomously provide care to patients, and all the content and behavior are based on the locally validated clinical pathway. All participants provided informed consent to be included in each care journey, ensuring their voluntary participation.

### Endpoints and Variables

The primary aim was to study the perceived usefulness of this PDCP captured through a questionnaire administered to HCPs piloting UpHill Route v3. This questionnaire was developed so that the constructs under each question contributed to understanding the perceived usefulness of UpHill Route v3: (1) adherence to clinical protocols (ease of compliance with protocols; (2) clinical decision support (measure of recommendation relevance); (3) improvement in patient care (perceived value of the presented recommendations to the patient); (4) task management (time-saving benefits); and (5) confidence in patient management (reliability of support for delivery of exhaustive and proper care). Each question was answered using a Likert scale ranging from 0 to 10, indicating the participant’s level of agreement with the presented component concerning PDCP usefulness. The questionnaire was written in Portuguese and distributed to participants via email. It is available in the supplementary materials (Multimedia Appendix 1).

The secondary aim was to describe the number and type of decisions supported by the software, by analyzing human-computer interactions made on the UpHill Route v3 graphical user interface. Decisions made with the support of the software are encapsulated in decision gateways, which are elements within UpHill Route v3 that prompt decision-making based on specific criteria. These criteria may be related to the patient’s condition, signs and symptoms, or clinical results. Multimedia Appendix 2 represents an example interface for UpHill Route v3 graphical interface for the type 2 diabetes mellitus clinical pathway of a test patient. In this interface, HCPs are presented with patient-specific actions and decisions in the center panel. On the right panel, supportive complementary information related to the selected action or decision in the central panel is presented to the HCP. Data were collected from user interactions with UpHill Route v3, as well as from its internal database. Participants were characterized according to demographic variables (eg, sex, age) and years of clinical experience. Additionally, information on academic activity as a binary variable indicating whether participants were involved in academic roles alongside their clinical duties was collected.

### Data Sources/Measurement

Primary objective data were collected through an online questionnaire. The questionnaire (in Portuguese) was delivered through Microsoft Forms and sent to the participants through email and are available in Multimedia Appendix 1. The analysis of the data was done using Microsoft Excel and STATA (version 14; StataCorp).

Secondary objective data were collected through queries from UpHill Route v3 internal database.

### Data Processing Protocol

Data were collected from the software’s database to describe the number and type of decisions supported by the software. The database recorded each option clicked on by the user for each pathway element. Then, these data were aggregated by pathway elements (ie, actions and decisions), each with a unique identification number. To analyze these elements and their selected options, queries on the database were performed, aggregating the same pathway element across all patient records.

Decisions were categorized into one of the ten clinical decision types as defined in the Decision Identification and Classification Taxonomy for Use in Medicine (DICTUM) [10]. These includes gathering additional information, evaluating test results, defining problems, drug-related decisions, therapeutic procedure-related decisions, legal and insurance-related decisions, contact-related decisions, advice, and precaution, setting treatment goals, and deferment.

Actions were categorized as clinical if assigned to nurses or physician profiles, or as nonclinical if assigned to administrative profiles. For example, clinical tasks included the recommendations to request a mandatory diagnostic test, while nonclinical tasks involved appointment scheduling. All data were aggregated by pathway elements, combining all the patient records without any identifiable patient variable.

### Study Size

For the primary objective, all 48 potentially eligible HCPs who were actively piloting UpHill Route v3 at the time of study recruitment were invited via email to answer the questionnaire.

For the secondary objectives, internal data were retrieved anonymously for HCPs and patient data. Therefore, data from the activities of all 48 professionals was analyzed.

### Statistical Methods

Descriptive statistics were used to summarize the demographic characteristics of the participants. Continuous variables were summarized with central tendency and dispersion measures, namely mean and standard deviation. Categorical variables were reported as relative and absolute frequencies. Bivariate analysis was conducted with a *χ*^2^ test for categorical variables and a 2-tailed Student *t* test for continuous variables.

To address the primary objective, responses collected from the questionnaire were analyzed by calculating mean scores and measures of dispersion (standard deviation) for each question. Higher mean scores indicated greater perceived usefulness of UpHill Route v3, while low dispersion reflected stronger agreement among participants regarding the software’s usefulness.

To address the secondary objective, we categorized and counted the types of decisions involving human-computer interaction, which were presented as absolute and relative frequencies. Additionally, we analyzed the tasks suggested by UpHill Route v3 within each care pathway, reporting these tasks as percentages.

### Ethical Considerations

This research project was submitted and approved by NOVA Medical School under the main author’s PhD research project “Characterizing uncertainty in decision making with complex multimorbidity patients,” (No.108/2021/CEFCM) (Multimedia Appendix 3). The study involved secondary analysis of existing data for which informed consent was obtained at the time of initial collection, and the original consent and institutional review board approval covered secondary analysis without requiring additional consent from the participants. All data used in this study were fully anonymized to ensure privacy and confidentiality. No compensation was provided to participants.

## Results

A total of 22 participants answered the questionnaire (response rate, 46%). The mean age of the participants was 44.7 (SD 10.6) years, while mean years of experience was 20.6 (SD 11.0) years. Of the 22 participants, 15 (68%) were female; 9 (41%) were medical doctors, and the remaining 13 (59%) were nurses. Additionally, 2 (9%) of participants were involved in academic activities.

Bivariate analysis showed no significant associations between gender and medical doctor status (*P*=.30), gender and nurse status (*P*=.13), or gender and involvement in academic activities (*P*=.66). Similarly, *t* tests for continuous variables indicated no significant differences in age (*P*=.54) or years of experience (*P*=.44) between male and female participants.

### Perceived Usefulness of UpHill Route V3

Participants rated the statement “UpHill Route v3 makes it easier to manage patients according to the clinical protocols of my institution” with a mean score of 8.06 (SD 1.73), a statement that aimed to assess the ability to easily comply with protocols. Focusing on clinical decision support, the statement “the UpHill Route v3 provides me with relevant clinical recommendations at the right time” received a mean score of 8.05 (SD 1.73). For the statement “the UpHill Route v3 helps me to improve my patient care”, which evaluated the sense of patient care improvement, the mean score was 7.63 (SD 2.22). Finally, for confidence in patient management, “the UpHill Route v3 gives me more confidence that my patients are being properly cared for” received a mean score of 8.26 (SD 1.56). The results of the questionnaire are presented in Table 1.

**Table 1.** Results of the questionnaire applied to HCPs^a^ to assess the perceived usefulness of the UpHill Route v3^b^.

Question	Mean score (SD)
UpHill Route v3 makes it easier to manage patients according to the clinical protocols of my institution	8.06 (1.73)
The UpHill Route v3 provides me with relevant clinical recommendations at the right time	8.05 (1.73)
The UpHill Route v3 helps me to improve my patient care	7.63 (2.22)
The UpHill Route v3 gives me more confidence that my patients are being properly cared for	8.26 (1.56)

a HCP: health care practitioner.

b Responses collected from the questionnaire were analyzed by calculating mean scores and measures of dispersion (standard deviation) for each question using the Likert scale ranging from 0 (do not agree) to 10 (totally agree) indicated the participant’s level of agreement with UpHill Route v3..

There were no significant differences in the perceptions of effectiveness of the UpHill Route v3 between physicians and nurses, as well as between genders.

Overall, the demographic characteristics or perceived usefulness of the UpHill Route v3 did not significantly differ based on gender or professional role (ie, medical doctor vs. nurse).

### Decisions Supported by the Software

Data were collected from heart failure, diabetes, and cancer (colorectal and breast) care pathways across the four different institutions (ie, LHUs).

Forty-eight HCPs used UpHill Route for supporting decisions of a total of 3574 patients, with 25,741 decisions captured. HCPs answered 427 decision gateways, performed 9254 actions, and the system generated 2429 clinical notes. Overall, there were 37,534 activities (ie, sum of decisions, actions, and notes) performed in UpHill Route v3. These results are summarized in Table 2.

**Table 2.** Aggregated data of human-computer interactions across care pathways.

Local health unit (Care pathway)	Patients (n=3574)	Gateways (n=427)	Decisions (n=25,741)	Actions (n=9254)	Clinical notes (n=2429)	Total activities per pathway, n (activities per patient)
Litoral Alentejano (heart failure)	609	76	8249	2570	0	10,819 (19.5)
Litoral Alentejano (multimorbidity)	668	207	10,680	2350	0	13,030 (17.8)
Coimbra (colorectal cancer)	355	3	685	1356	0	2041 (5.8)
São José (colorectal cancer)	202	25	883	856	144	1883 (9.3)
São José (breast cancer)	223	32	878	1079	107	2064 (9.3)
Amadora/Sintra (diabetes)	1517	84	4466	1053	2178	7697 (5.1)
Total	—^a^	—	—	—	—	37,534 (10.5)

a Not applicable.

The Litoral Alentejano multimorbidity care pathway presented the highest number of activities per patient, with an average of 19.5 activities, followed by the Litoral Alentejano heart failure care pathway which exhibited a high level of clinical activity, with 17.8 activities per patient. São José colorectal and breast cancer pathways had an average of 9.3 activities per patient. Coimbra colorectal cancer and Amadora/Sintra diabetes care pathways averaged 5.8 and 5.1 activities per patient, respectively. Overall, the mean number of clinical activities per patient performed in the four health institutions using the UpHill Route v3 was 10.5.

As part of the secondary objective, the categorization of clinical decisions showed that the majority of decisions (9215/25,841, 35.7%) were related to “defining problem”, followed by “gathering additional information” (n=5759, 22.3%) and “treatment goal” (n=4845, 18.7%). The distribution of these decisions varied across different care pathways, with Litoral Alentejano multimorbidity and heart failure accounting for the largest proportions, representing 41.3% (n=10,678) and 31.9% (n=8251) of the total decisions, respectively. Other decision types, such as “contact-related” and “therapeutic procedure–related,” accounted for smaller proportions, 7.0% (n=1814) and 5.0% (n=1280), respectively. This distribution highlights the diverse range of decision types supported by UpHill Route v3, tailored to the specific needs of different clinical contexts. The results of the decision categorization, presented by clinical journey are summarized in Multimedia Appendices 4 and 5 .

While analyzing decision types, a total of 1814 (n=7.0%) decisions were found to be contact-related. These involved admitting or discharging patients, scheduling follow-ups, and referring patients to other parts of the health care system. There were 460 (1.8%) deferment decisions, which involved actively delaying or rejecting a decision on a problem presented by the patient. The highest number of decisions were defining-problem decisions, totaling 9215/25,841 (35.7%). These complex, interpretative assessments defined what the problem was, including diagnostic conclusions, evaluations of health status, etiological inferences, and prognostic judgments. A total of 1415 (5.5%) decisions were related to drug therapy, which involved starting, stopping, altering, or maintaining a drug regimen. There were 1053 (4.1%) decisions related to the evaluation of test results, involving simple, normative assessments of clinical findings and tests. A total of 5759 (22.3%) decisions were aimed at gathering additional information, which involved obtaining information from sources other than patient interviews, physical examinations, and patient charts. There were 1280 (5.0%) decisions related to therapeutic procedures; these involve planning, performing, or refraining from therapeutic interventions. A total of 4845 (18.7%) decisions were related to setting treatment goals, which involved defining specific objectives for patient care. The comprehensive details of each decision type’s categorization can be found in Multimedia Appendix 6.

## Discussion

### Principal Findings

This study evaluated the perceived usefulness and practical implementation of the UpHill Route v3 as a PDCP in various clinical settings. The primary outcome focused on the perceived usefulness of the system, as assessed by HCPs through a structured questionnaire. The results demonstrated high scores across multiple dimensions, including adherence to clinical protocols, clinical decision support, and confidence in patient management. Specifically, participants rated the system highly in the ease of adhering to clinical protocols by providing relevant clinical recommendations, demonstrating that HCPs perceive UpHill Route v3 as a valuable tool in clinical settings. Our analysis found no significant differences in the perceived usefulness of UpHill Route v3 between genders or professional roles (medical doctors vs nurses). This lack of disparity indicates that UpHill Route v3 is perceived to be equally beneficial across different demographic and professional groups within health care settings. Overall, this posits that the software with its decision support capabilities proves valuable in adhering to clinical guidelines, providing relevant clinical guidance, enhancing patient care, and instilling confidence in the provided care.

The high scores across multiple dimensions of perceived usefulness indicate that HCPs find the UpHill Route v3 system valuable for clinical decision support. The system’s ability to integrate clinical protocols and provide timely recommendations appears to enhance both clinical efficiency and the quality of patient care.

Concerning secondary outcomes, the data indicated significant usage of clinical activities being supported by UpHill Route v3, averaging 10.5 activities per patient. There were expected variations in clinical activities per patient across different care pathways. Litoral Alentejano multimorbidity and heart failure cases required the most activities per patient, suggesting highly complex pathways with numerous decision points. These care pathways naturally require more complex clinical activity for the management of these conditions. In contrast, Coimbra Colorectal Cancer and Amadora Sintra Diabetes involved fewer activities per patient, reflecting a more focused and shorter clinical pathway in those users. The distribution of decisions supported by UpHill Route v3 varied significantly, with defining–problem decisions and gathering additional information being the most common. This finding is consistent with the complex nature of clinical diagnostics and the need for comprehensive information gathering to inform clinical decisions in the follow-up phase of symptomatic chronic diseases such as heart failure or multimorbidity [11]. The system’s ability to support these critical decision types highlights its relevance and utility in clinical practice. Overall, the categorization of decisions and tasks demonstrates the system’s versatility in supporting various aspects of patient management, from diagnosis and treatment to follow-up care. These findings suggest that the UpHill Route v3 system can effectively bridge the gap between clinical guidelines and real-world practice, potentially leading to improved patient management.

The study’s findings on UpHill Route v3 align with existing literature on the effectiveness of CDSS in healthcare [12]. Our findings emphasize the importance of CDSS, particularly PDCP, in providing timely and accurate decision support, through personalization and longitudinal continuity, which is crucial for clinicians in complex and dynamic environments [13]. This aligns with findings from multiple systematic reviews and studies. Although many CDSS enhance practitioner performance, evidence of their impact on patient outcomes remains limited and inconsistent, indicating a need for further research[14]. A recently published systematic review and meta-analysis focused on the primary prevention of cardiovascular disease demonstrated that CDSS were effective in improving blood pressure clinical target attainments. However, multilayered barriers affecting the uptake, longer-term use and active engagement from clinicians and patients may hinder their full potential in achieving other quality-of-care outcomes [15]. While digital care pathways have shown promise in optimizing the efficiency of care delivery, effective implementation requires training, resource allocation, support for information management, and optimizing alert algorithms to manage information overload [16,17].

The positive feedback on UpHill Route v3’s decision support capabilities reinforces the concept that a PDCP as a CDSS can enhance clinical decision-making and patient management, and the subsequent step for PDCPs is likely to be task automation of predictable and mandatory tasks. Previous findings in the literature highlight its benefits. For instance, a systematic review by Jones et al [18] highlighted how task automation through CDSS can streamline workflows, reduce administrative burdens, and minimize human errors, allowing HCPs to focus more on direct patient care.

Future research should explore the long-term impacts of task automation on clinical outcomes and examine how the integration of PDCPs with other health care technologies can provide efficient orchestration of patient health care.

Several limitations and potential sources of bias need to be acknowledged in this study. First, only 22 HCPs replied to the questionnaire, a small number that may limit the generalizability of the findings, although they worked at multiple health care centers. Additionally, the study relied on self-reported data to assess the perceived usefulness of the system, which could be subject to response bias. Despite anonymization, participants may have provided favorable responses due to social desirability or perceived expectations of the study organizers. However, a strength of the study is the inclusion of aggregated data from diverse patient populations (eg, heart failure, diabetes, breast and colorectal cancer) across various clinical settings (ie, primary and secondary care) and contexts (ie, urban and rural).

Although all authors are affiliated with UpHill Health, S.A., the study design, data collection, and analysis were conducted using standardized methodologies to ensure objectivity. The findings were interpreted transparently and align with prior research on clinical decision support systems, reinforcing their validity.

While the study’s findings are promising, the small sample size limits the generalization of the results. Future research should aim to include a larger and more diverse cohort across multiple health care settings (ie, various specialties and practice settings) and geographic regions to enhance external validity and applicability to broader clinical contexts. We speculate that variations in health care system infrastructure, clinical workflows, cultural factors, and user experience levels may influence the adoption and perceived effectiveness of PDCP.

Additionally, this study primarily focuses on short-term implementation outcomes. Future research should adopt a longitudinal approach to evaluate sustained adherence to protocols, patient outcomes, and cost efficiency, allowing for a more comprehensive assessment of the long-term impact of the UpHill Route v3 system on clinical outcomes and health care efficiency. Despite these limitations, the positive qualitative feedback and the objective quantitative interaction with the software by HCPs suggest that the UpHill Route v3 system is a valuable tool in various clinical settings, supporting decision-making and enhancing patient care.

### Conclusion

This study demonstrates that HCPs highly value PDCPs as CDSSs for enhancing adherence to clinical protocols, providing timely recommendations, and improving patient care. The system showed significant activity, with several decisions supported across various clinical journeys and settings.

Despite the study’s small sample size, the anonymous nature of the questionnaire and objective secondary outcome data provide robust evidence of the system’s effectiveness across different clinical scenarios such as chronic, oncological, and surgical conditions. Further research with larger and more diverse populations is needed to confirm these findings and explore the system’s long-term impact.

In summary, the PDCP systems support clinical decision-making and operational efficiency and promote the accurate execution of a wide range of clinical activities in patient management in health care settings.

## Supplementary material

10.2196/68581 Multimedia Appendix 1 Health Care Professionals Questionnaire.

10.2196/68581 Multimedia Appendix 2 UpHill Route V3 graphical interface.

10.2196/68581 Multimedia Appendix 3 Ethics committee approval.

10.2196/68581 Multimedia Appendix 4 Decision categorisation presented by care pathway.

10.2196/68581 Multimedia Appendix 5 Aggregated clinical pathway supported decisions categorized as by DICTUM taxonomy.

10.2196/68581 Multimedia Appendix 6 Table with detailed information on decision categorization presented by care pathway.

## Abbreviations

CDSS: clinical decision support systems
EHR: electronic health record
HCP: health care professional
PDCP: personalized digital care pathway
