# Supplementary material for: Personalized Digital Care Pathways Enable Enhanced Patient Management as Perceived by Health Care Professionals: Mixed-Methods Study
Source: JMIR Hum Factors. 2025 May 15;12:e68581. doi: 10.2196/68581 (PMC12097650; doi:10.2196/68581)
Supplement: Multimedia Appendix 1 [file humanfactors-v12-e68581-s001.docx]

# **Health Care Professional Questionnaire**

## Usefulness and satisfaction in using UpHill technology in your clinical activity.

Thank you for your willingness to take part in this study. It will only take about 5 minutes of your time.

Consent: This study aims to understand the perceived usefulness of a clinical decision support system, validated according to local clinical protocols, for patient management.

To do this, we ask you to complete a short questionnaire (you will answer in less than 5 minutes) which includes 4 general questions about you and 5 questions about your experience with UpHill. No identifying information will be collected.

This study is conducted by David Rodrigues, UpHill's Medical Director. If you would like to receive more information or contact the researcher directly, please write an email to david.rodrigues@nms.unl.pt.

Please answer the following questions honestly and to the best of your ability.

| UpHill’s Route Usefulness |  |
| --- | --- |
| 1. I have read the consent information, I have been given contact details to ask questions about it and at this point I have no doubts and want to go ahead.  I understand that by clicking on the ‘Yes’ button followed by ‘Next’ I am voluntarily consenting to take part in the study ‘Usefulness and satisfaction in the use of UpHill technology in your clinical activity’. | Yes/No |
| 2. Enter your age: | Numerical continuous |
| 3.Gender | Female/ Male / Other / Prefer not to say |
| 4.How many years of clinical experience do you have? | Numerical continuous |
| 5.Are you currently involved in academic activity? | Yes/ No |
| 6. UpHill Route v3 makes it easier to manage patients according to the clinical protocols of my institution | (Likert scale from 0 – Do not Agree to 10 – Totally agree) |
| 7. The UpHill Route v3 provides me with relevant clinical recommendations at the right time | (Likert scale from 0 – Do not Agree to 10 – Totally agree) |
| 8. The UpHill Route v3 helps me to improve my patient care | (Likert scale from 0 – Do not Agree to 10 – Totally agree) |
| 9. The UpHill Route v3 gives me more confidence that my patients are being properly cared for | (Likert scale from 0 – Do not Agree to 10 – Totally agree) |

Thank you for your collaboration.
