# Supplementary material for: Personalized Digital Care Pathways Enable Enhanced Patient Management as Perceived by Health Care Professionals: Mixed-Methods Study
Source: JMIR Hum Factors. 2025 May 15;12:e68581. doi: 10.2196/68581 (PMC12097650; doi:10.2196/68581)
Supplement: Multimedia Appendix 2 [file humanfactors-v12-e68581-s002.docx]

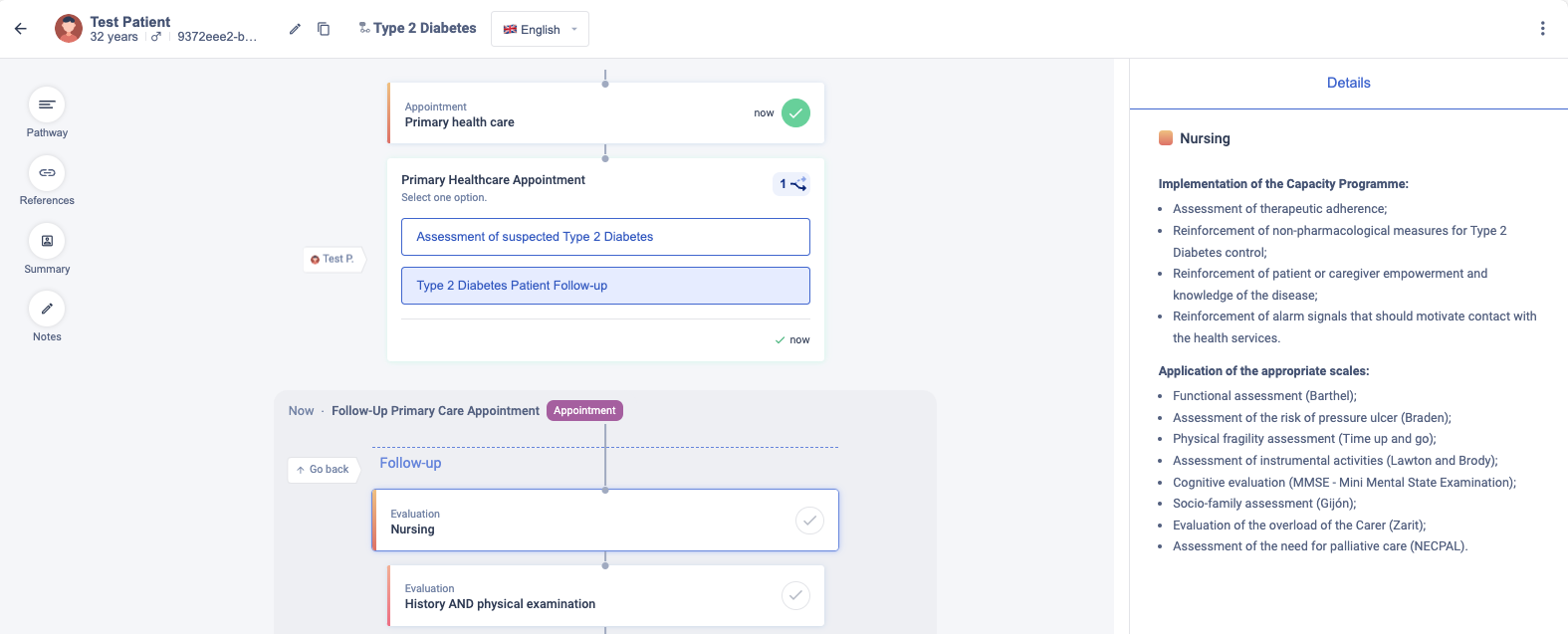


UpHill Route v3 graphical interface presenting a completed appointment action, a decision gateway for the type of appointment and the suggested evaluations actions for a Type 2 Diabetes patient in a primary care context.
