# Supplementary figures and images for: Personalized Digital Care Pathways Enable Enhanced Patient Management as Perceived by Health Care Professionals: Mixed-Methods Study
Source: JMIR Hum Factors. 2025 May 15;12:e68581. doi: 10.2196/68581 (PMC12097650; doi:10.2196/68581)

# **Ethics Committee**

#
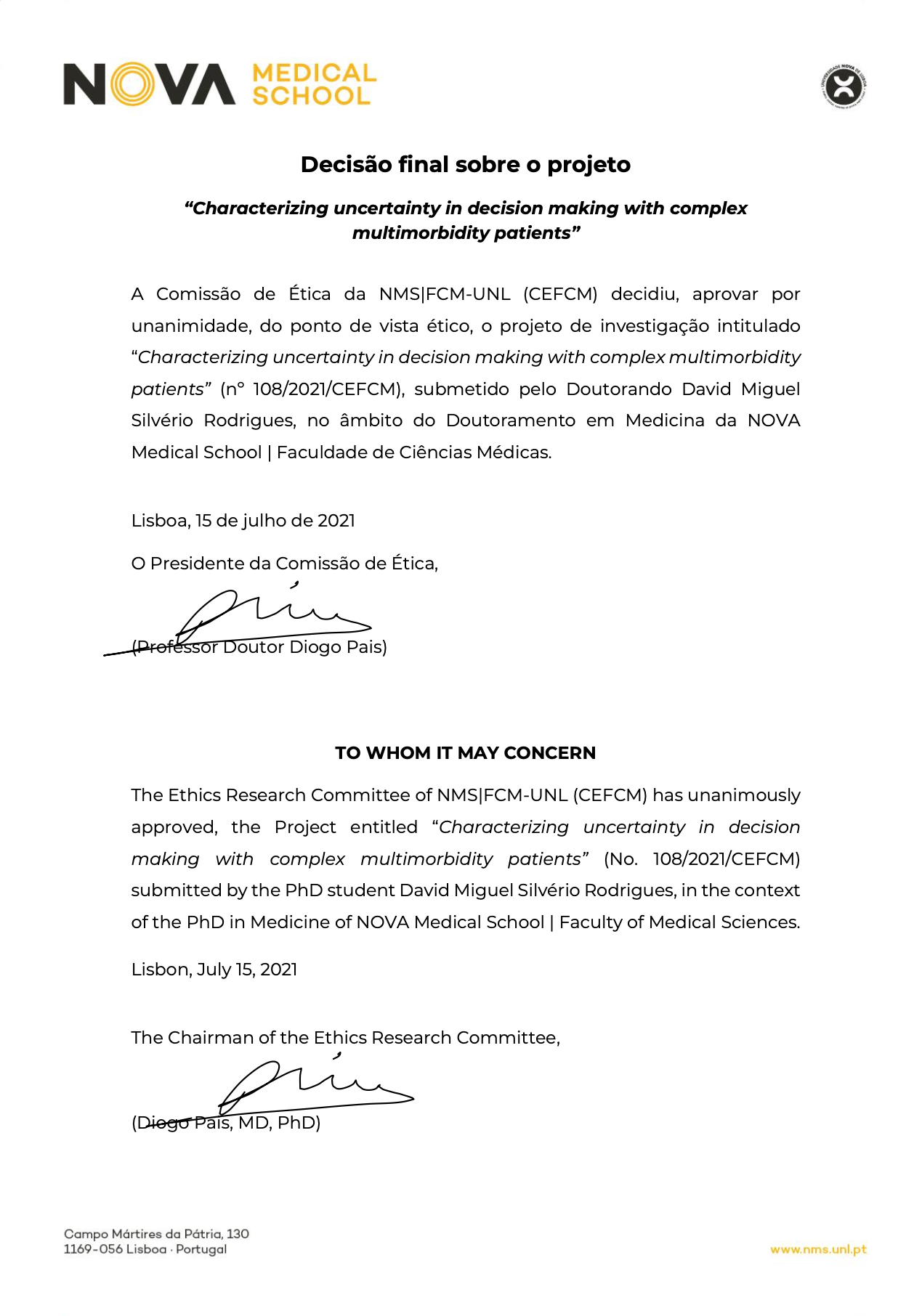

Supplement: Multimedia Appendix 3 [file humanfactors-v12-e68581-s003.docx]

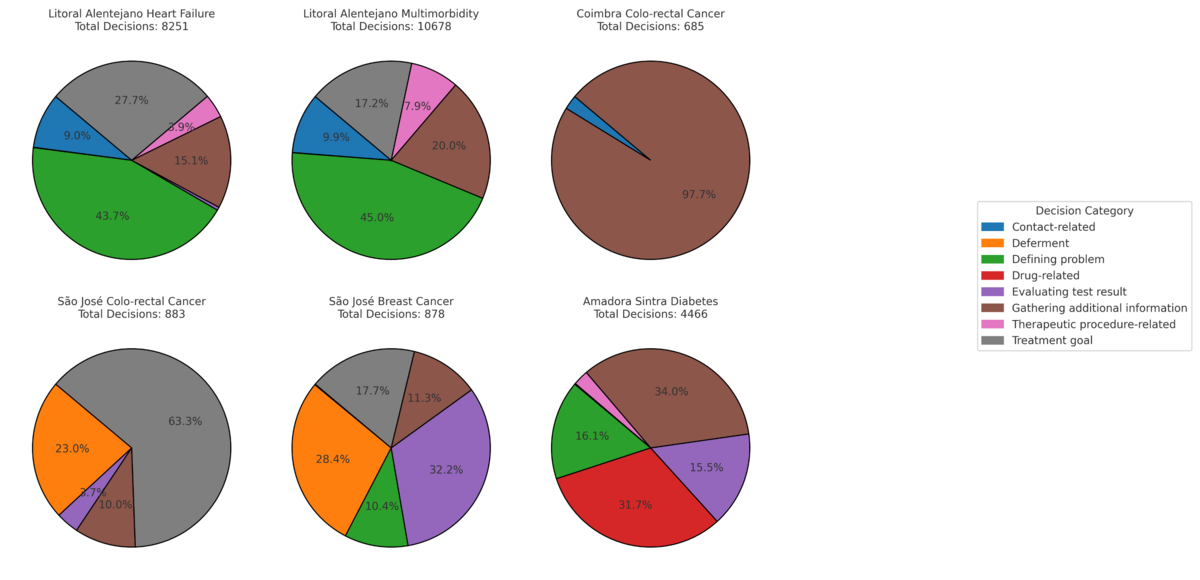

Supplement: Multimedia Appendix 4 [file humanfactors-v12-e68581-s004.png]
