# Supplementary material for: Personalized Digital Care Pathways Enable Enhanced Patient Management as Perceived by Health Care Professionals: Mixed-Methods Study
Source: JMIR Hum Factors. 2025 May 15;12:e68581. doi: 10.2196/68581 (PMC12097650; doi:10.2196/68581)
Supplement: Multimedia Appendix 5 [file humanfactors-v12-e68581-s005.docx]

**Aggregated clinical pathway supported decisions categorized as by DICTUM taxonomy**

| **Decision type** | **Sum of Total clicks** | |
| --- | --- | --- |
| **Contact-related** | **1814** | |
| Appointment: | 8 | |
| Heart failure hospital appointment | 419 | |
| Multimorbidity Hospital Consultation | 378 | |
| Primary Healthcare Appointment | 318 | |
| Referral criteria? | 674 | |
| What is the most appropriate referral option? | 17 | |
| **Deferment** | **460** | |
| Is the patient able to proceed to Multidisciplinary Consultation? | 452 | |
| Patient referred to: | 8 | |
| **Defining problem** | **9215** | |
| Advanced Heart Failure criteria | 706 | |
| Any signs of clinical decompensation? | 54 | |
| Choose the best approach: | 23 | |
| Clear signs of clinical decompensation? | 116 | |
| Clinical instability criteria | 1616 | |
| Do you still have a clinical suspicion of Heart Failure? | 1 | |
| Does the patient have alarm signs? | 1657 | |
| Does the patient show signs of clinical decompensation that warrant further evaluation? | 1207 | |
| Heart Failure Classification | 2 | |
| Need for face-to-face consultation? | 18 | |
| Need to proceed to telephone clinical evaluation? | 64 | |
| No evidence of active disease? | 2 | |
| Patient presents tumor: | 63 | |
| Patient with an indication for hospital referral? | 2 | |
| Patient with previous heart attack or stroke? | 719 | |
| Patient with: | 2932 | |
| Signs of decompensation/doubts? | 30 | |
| Surgery? | 3 | |
| **Drug-related** | **1415** | |
| Does the patient have any indication to take insulin? | 1 | |
| Patient on insulin? | 764 | |
| Patient with an indication to start a statin? | 650 | |
| **Evaluating test result** | **1053** | |
| Albumin results? | 103 | |
| Are further tests necessary? | 224 | |
| Are there echocardiographic alterations that confirm the diagnosis of HF? | 2 | |
| Do you have access to natriuretic peptides for immediate execution in point-of-care modality? | 21 | |
| Does the patient have analyses scheduled for next month? | 13 | |
| Genomic platform result: | 15 | |
| HbA1c results? | 240 | |
| Is it necessary to assess genetic markers? | 33 | |
| LDL results? | 98 | |
| Need for a genomics platform? | 44 | |
| NT Pro-BNP ≥125 pg/mL? | 19 | |
| Patient referred from Primary Healthcare AND/OR with investigation with complementary exams already performed? | 1 | |
| TFG results? | 240 | |
| **Gathering additional information** | **5759** | |
| Admission criteria: | 51 | |
| Any alarm sign mentioned by the patient? | 559 | |
| Did the patient indicate the start and end dates of the radiotherapy treatment? | 21 | |
| Discharge criteria confirmed? | 680 | |
| Do the symptoms warrant: | 9 | |
| Do you want to proceed to pre-discharge assessment? | 421 | |
| Exclusion criteria for follow-up by Hilly? | 2233 | |
| Patient with eligibility criteria for use-case? | 1518 | |
| Symptoms justify observation? | 267 | |
| **Therapeutic procedure-related** | **1280** | |
| Assessment of hospitalization criteria: | 673 | |
| Need to refer to Emergency Room? | 55 | |
| Patient followed for dyslipidemia control? | 112 | |
| Patient with optimised therapy and stabilised? | 440 | |
| **Treatment goal** | **4845** | |
| Are you sure you want to proceed with discharge? | 10 | |
| Choose the plan for your patient: | 172 | |
| Criteria for admission to the Intermediate Care Unit? | 1 | |
| Criteria for keeping follow-up in Primary Healthcare? | 802 | |
| Criteria for referral to Diabetes Hospital Consultation? | 320 | |
| Criteria for referral to Heart Failure Hospital Consultation? | 776 | |
| Criteria for referral to Pneumology Hospital Consultation? | 118 | |
| Criteria for referral to the Emergency Service? | 82 | |
| Criteria for transition to Primary Healthcare? | 831 | |
| Decision on the initial approach: | 130 | |
| Do you want to keep the patient in breast pathology follow-up? | 6 | |
| Do you wish to keep the patient in a follow-up appointment for colorectal pathology? | 6 | |
| Does the patient need to be referred to any of the following areas? | 172 | |
| Initial approach: | 127 | |
| Is it necessary to change the usual plan? | 6 | |
| Medical decision: | 1 | |
| Palliative Care criteria | 715 | |
| Plan: | 19 | |
| Stability at home criteria: | 469 | |
| Treatment: | 50 | |
| What is the next plan for the patient? | 32 | |
| **Grand Total** | | **25841** |
