# Supplementary material for: Personalized Digital Care Pathways Enable Enhanced Patient Management as Perceived by Health Care Professionals: Mixed-Methods Study
Source: JMIR Hum Factors. 2025 May 15;12:e68581. doi: 10.2196/68581 (PMC12097650; doi:10.2196/68581)
Supplement: Multimedia Appendix 6 [file humanfactors-v12-e68581-s006.docx]

| **Decision categorisation presented by care pathway** | | | | | | | |
| --- | --- | --- | --- | --- | --- | --- | --- |
| **Decision type** | **Litoral Alentejano Heart Failure** | **Litoral Alentejano Multimorbidity** | **Coimbra Colo-retal cancer** | **São José Colo-rectal cancer** | **São José Breast cancer** | **Amadora Sintra Diabetes** | **Total (%)** |
| Contact-related | 744 | 1,053 | 16 | 0 | 1 | 0 | 1,814 (7.0%) |
| Deferment | 4 | 4 | 0 | 203 | 249 | 0 | 460 (1.8%) |
| Defining problem | 3,603 | 4,800 | 0 | 0 | 91 | 721 | 9,215 (35.7%) |
| Drug-related | 0 | 0 | 0 | 0 | 0 | 1,415 | 1,415 (5.5%) |
| Evaluating test result | 43 | 0 | 0 | 33 | 283 | 694 | 1,053 (4.1%) |
| Gathering additional information | 1,249 | 2,136 | 669 | 88 | 99 | 1,518 | 5,759 (22.3%) |
| Therapeutic procedure-related | 321 | 847 | 0 | 0 | 0 | 112 | 1,280 (5.0%) |
| Treatment goal | 2,287 | 1,838 | 0 | 559 | 155 | 6 | 4,845 (18.7%) |
| Total (%) | 8,251 (31.9%) | 10,678 (41.3%) | 685 (2.6%) | 883 (3.4%) | 878 (3,4%) | 4,466 (17.2%) | 25,841 |
